# Caffeine inhibits TGFβ activation in epithelial cells, interrupts fibroblast responses to TGFβ, and reduces established fibrosis in *ex vivo* precision-cut lung slices

**DOI:** 10.1136/thoraxjnl-2015-208215

**Published:** 2016-02-24

**Authors:** Amanda L Tatler, Josephine Barnes, Anthony Habgood, Amanda Goodwin, Robin J McAnulty, Gisli Jenkins

**Affiliations:** 1 Division of Respiratory Medicine, Nottingham City Hospital, University of Nottingham, Nottingham, UK; 2 UCL Respiratory Centre for Inflammation and Tissue Repair, University College London, London, UK

**Keywords:** Idiopathic pulmonary fibrosis

## Abstract

Caffeine is a commonly used food additive found naturally in many products. In addition to potently stimulating the central nervous system caffeine is able to affect various systems within the body including the cardiovascular and respiratory systems. Importantly, caffeine is used clinically to treat apnoea and bronchopulmonary dysplasia in premature babies. Recently, caffeine has been shown to exhibit antifibrotic effects in the liver in part through reducing collagen expression and deposition, and reducing expression of the profibrotic cytokine TGFβ. The potential antifibrotic effects of caffeine in the lung have not previously been investigated. Using a combined in vitro and ex vivo approach we have demonstrated that caffeine can act as an antifibrotic agent in the lung by acting on two distinct cell types, namely epithelial cells and fibroblasts. Caffeine inhibited TGFβ activation by lung epithelial cells in a concentration-dependent manner but had no effect on TGFβ activation in fibroblasts. Importantly, however, caffeine abrogated profibrotic responses to TGFβ in lung fibroblasts. It inhibited basal expression of the α-smooth muscle actin gene and reduced TGFβ-induced increases in profibrotic genes. Finally, caffeine reduced established bleomycin-induced fibrosis after 5 days treatment in an ex vivo precision-cut lung slice model. Together, these findings suggest that there is merit in further investigating the potential use of caffeine, or its analogues, as antifibrotic agents in the lung.

Caffeine (1,3,7-tri-methylxanthine) is one of the most commonly consumed food additives worldwide and has wide-ranging pharmacological activities including effects on the central nervous, cardiovascular and respiratory systems. It can act as an antagonist of adenosine receptors, an inhibitor of phosphodiesterases and an activator of ryanodine receptors. Caffeine is similar in structure and function to theophylline and can improve lung function in asthmatics by inducing bronchodilation.^1^ Furthermore, caffeine citrate (Calficit) is clinically approved and commonly used to treat bronchopulmonary dysplasia and apnoea in premature infants.^2^ In recent years, caffeine has been shown to exhibit antifibrotic effects in the liver. Consumption of caffeine, often in the form of coffee, is associated with reduced hepatic fibrosis in patients suffering from chronic hepatitis C virus infection.^3^ In in vivo animal models of liver fibrosis caffeine can reduce collagen deposition and collagen mRNA,^4^ ^5^ and can block expression of the profibrotic cytokine TGFβ.^6^ Furthermore, caffeine can inhibit profibrotic responses in hepatic stellate cells, the key effector cell in the development of liver fibrosis.^7^ Despite the known effects of caffeine on lung function the potential antifibrotic effects of caffeine in the lung have not previously been investigated.

In the present study we have used a combination of in vitro cell and ex vivo tissue approaches to investigate the hypothesis that caffeine can inhibit fibrogenesis in the lung. The pathogenesis of pulmonary fibrosis is thought to involve activation of TGFβ by lung epithelial cells following repeated microinjury to the epithelium, causing transdifferentiation of fibroblasts into profibrotic myofibroblasts, which ultimately leads to deposition of extracellular matrix within the lung interstitium and deteriorating lung function and/or death. We have demonstrated that caffeine inhibits endogenous TGFβ activation in immortalised human bronchial epithelial cells in a concentration-dependent manner by measuring levels of phosphorylated Smad2 (P-Smad2) (figure 1A). Furthermore, basal levels of the TGFβ-induced gene *PAI1* were inhibited by caffeine over 24 h (figure 1B). Importantly, the effect of caffeine on TGFβ activation was specific to epithelial cells since caffeine had no effect on endogenous TGFβ activation in lung fibroblasts isolated from either non-fibrotic control (NL) donors or patients with idiopathic pulmonary fibrosis (IPF) (figure 1C). Caffeine did not affect cell viability in any cell type tested (see online supplementary figures S1B–D).[Supplementary-material SM1]

10.1136/thoraxjnl-2015-208215.supp1 Supplementary data

10.1136/thoraxjnl-2015-208215.supp3 Supplementary figure

**Figure 1:**
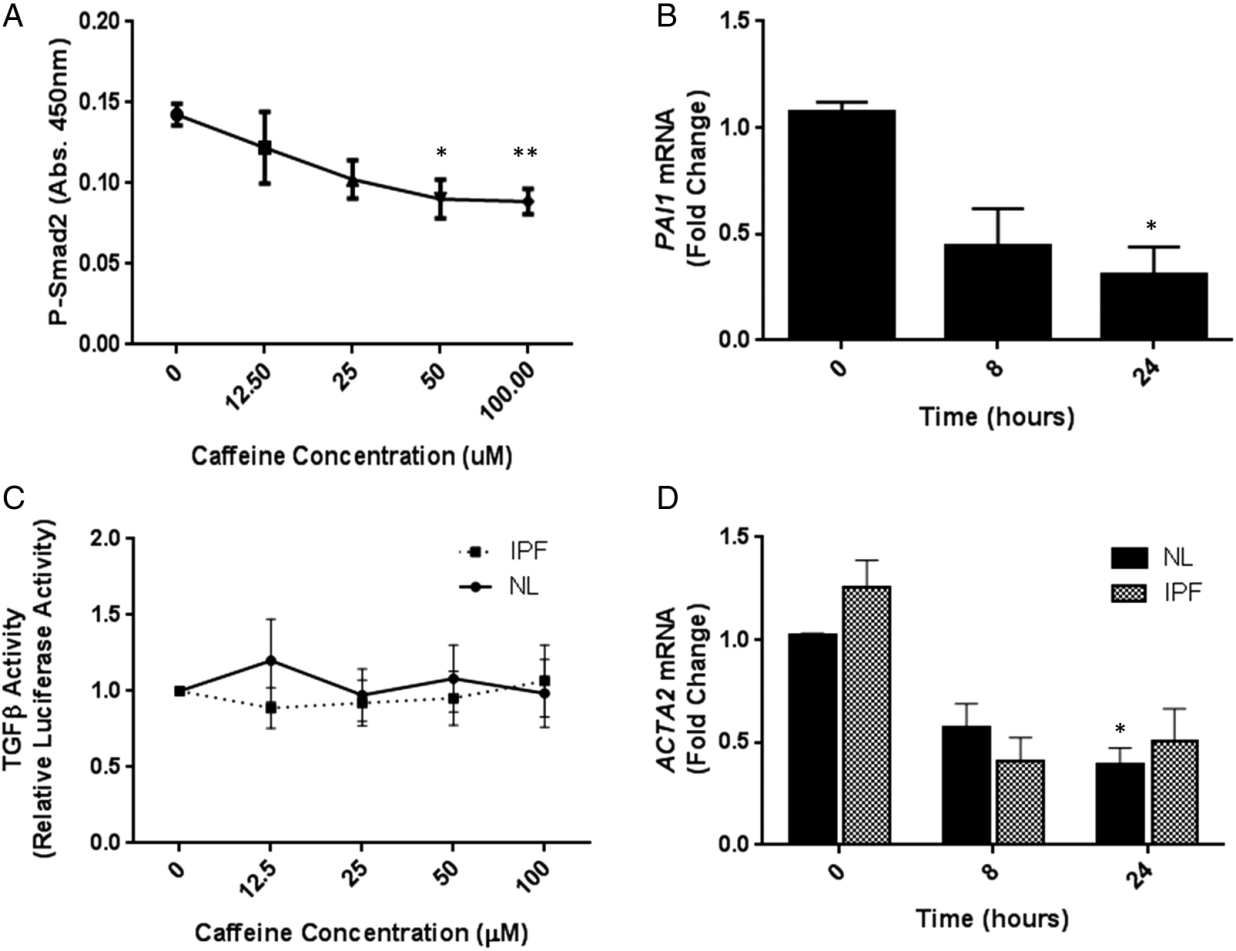
(A) Immortalised human bronchial epithelial cells (iHBECs) were stimulated with increasing concentrations of caffeine for 4 hours and PSmad2 levels measured. Figure shows mean data±SEM from three independent experiments. (B) iHBECs were stimulated with 50 µM caffeine and PAI1 mRNA levels measured. Data are expressed as mean fold change over control (0 h)±SEM from three independent experiments. (C) Non-fibrotic control (NL) and idiopathic pulmonary fibrosis (IPF) fibroblasts were stimulated with increasing concentrations of caffeine and TGFβ activation assessed by TMLC reporter assay. Figure shows mean data±SEM from n=3 NL and n=3 IPF donors. (D) NL and IPF fibroblasts were stimulated with 50 µM caffeine and ACTA2 mRNA levels measured. Data are expressed as mean fold change over control (0 h for NL or IPF, respectively)±SEM. Figure shows mean data from n=3 NL and n=3 IPF donors. *p<0.05 **p<0.01.

Increased expression of α-smooth muscle actin (α-SMA) in fibroblasts is a key marker of fibroblast-myofibroblast transdifferentiation. We therefore assessed the effect of caffeine on expression of α-SMA (*ACTA2*) transcript in NL and IPF fibroblasts as a surrogate for measuring fibroblast-myofibroblast transdifferentiation. Caffeine inhibited basal *ACTA2* mRNA expression in both diseased and control fibroblasts (figure 1D) suggesting that it can interrupt fibroblast-myofibroblast transdifferentiation, a key fibrogenic process.

To further investigate the effect of caffeine on profibrotic responses in lung fibroblasts we investigated caffeine's actions on TGFβ-induced gene expression. TGFβ increased expression of *PAI1*, *ACTA2 a*nd *TGFB1 i*n IPF fibroblasts after 24 h and caffeine abrogated these responses (figure 2A–C). This effect was also observed in NL fibroblasts (see online supplementary figures E–G). Together these in vitro data demonstrate that caffeine may inhibit fibrogenesis through concomitant, but distinct, actions on both epithelial cells and lung fibroblasts. It has previously been suggested that inhibition of PDE4 can mediate TGFβ-induced fibroblast to myofibroblast differentiation, therefore we hypothesised that caffeine's actions on TGFβ-induced fibroblast responses might be mediated via PDE4.^8^ To investigate this further we used the PDE4 inhibitor roflumilast. Treatment of IPF fibroblasts with 10 µM roflumilast did not recapitulate the effects of caffeine on TGFβ-induced *PAI1, ACTA2* and *TGFB1* mRNA expression (figure 2D–F). These data suggest the inhibitory effect of caffeine on TGFβ-induced gene expression is independent of its effects on PDE4.

**Figure 2:**
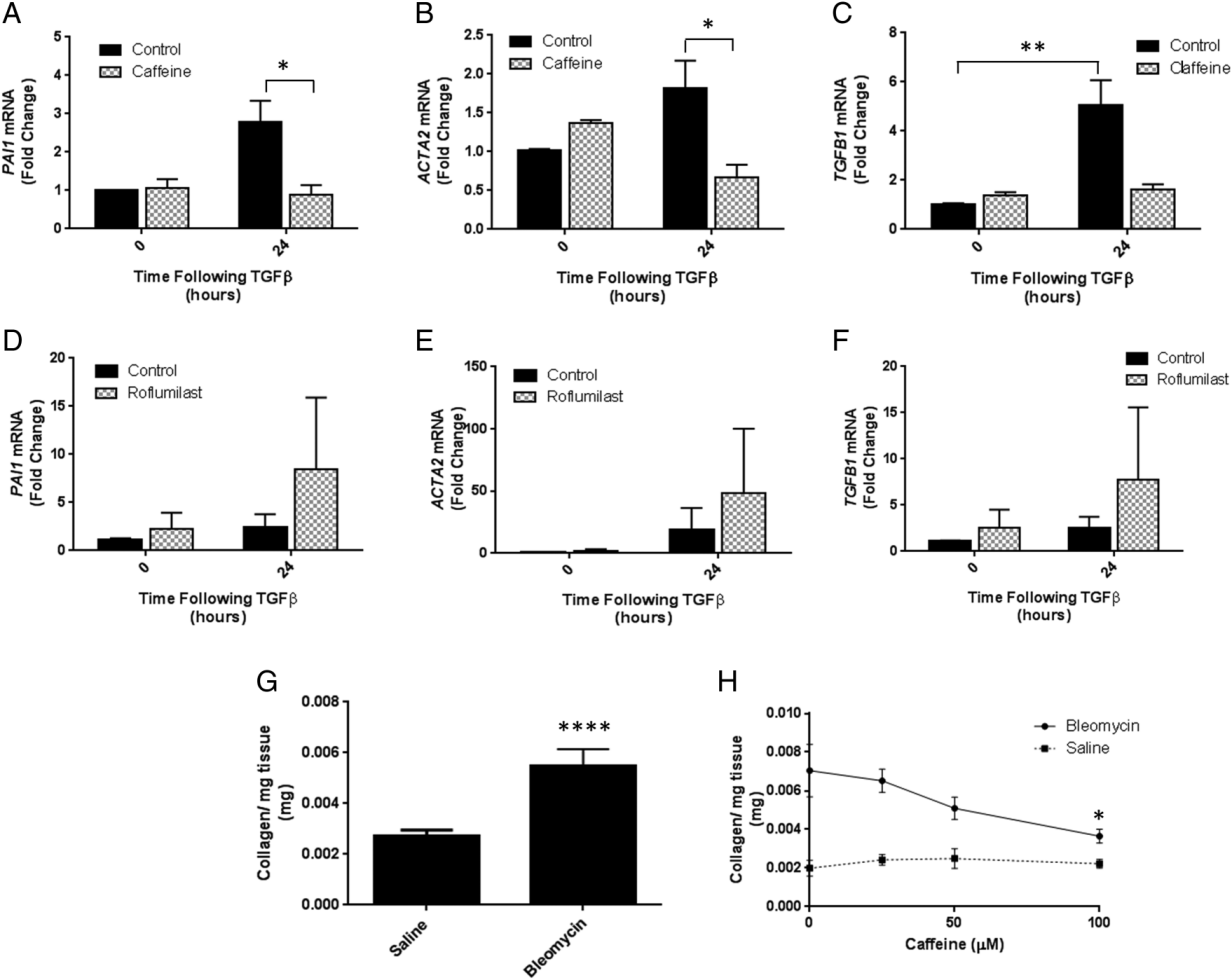
Idiopathic pulmonary fibrosis (IPF) fibroblasts were pretreated with 0 μM or 50 μM caffeine for 30 min then stimulated with 0 ng/mL or 2 ng/mL TGFβ for 24 h and (A) PAI1; (B) ACTA2; (C) TGFB1 gene expression measured. Data are expressed as mean fold change over control (0 h, 0 ng/mL TGFβ)±SEM from experiments performed on cells from three individual donors. IPF fibroblasts were pretreated with 0 μM or 10 μM roflumilast for 30 min then stimulated with 0 ng/mL or 2 ng/mL TGFβ for 24 h and (D) PAI1; (E) ACTA2; (F) TGFB1 gene expression measured. Data are expressed as mean fold change over control (0 h, 0 ng/mL TGFβ)±SEM from experiments performed on cells from four individual donors. (G) Precision-cut lung slices (PCLS) were prepared from the lungs of saline-treated and bleomycin-treated mice and collagen levels measured after 5 days in ex vivo culture. Data are expressed as mean collagen (mg) per mg of lung tissue±SEM from n=16 PCLS/group. (H) PCLS were prepared from the lungs of bleomycin-treated or saline-treated mice and treated for 5 days ex vivo with 0 μM, 25 μM, 50 μM and 100 μM caffeine. Data are expressed as mean collagen (mg) per mg of lung tissue±SEM from n=6 bleomycin PCLS or n=2 saline PCLS. *p<0.05, **p<0.01, ****p<0.0001.

Finally, to confirm that caffeine can act as an antifibrotic agent in the lung, we have used a novel ex vivo precision-cut lung slice (PCLS) model of pulmonary fibrosis. PCLS were prepared from mice treated with either bleomycin or saline control for 28 days and the presence of fibrosis determined using high-performance liquid chromatography. PCLS from bleomycin-treated animals contained significantly higher levels of collagen than PCLS from saline-treated animals (figure 2D). Crucially, caffeine was also able to significantly reduce collagen deposition in a concentration-dependent manner over 5 days within bleomycin-PCLS (figure 2E).

These data demonstrate for the first time that caffeine can act as an antifibrotic agent in the lung. Concentrations of caffeine achieved physiologically are generally less than 70 µM caffeine,^9^ furthermore, previous in vivo animal studies have shown antifibrotic effects in the liver with serum concentrations ranging from 38 µM to 59 µM.^4^ Our studies showed antifibrotic effects at concentrations between 50 µM and 100 µM supporting the hypothesis that physiological concentrations of caffeine may have antifibrotic effects in the lung. In the lung, caffeine appears to exhibit its antifibrotic effects through distinct actions on both epithelial cells and fibroblasts, which are two of the key effector cells involved in the pathogenesis of pulmonary fibrosis. It has previously been reported that caffeine is capable of interrupting TGFβ-induced Smad signalling in a lung epithelial cancer cell line.^10^ However, these data demonstrate that caffeine can also inhibit endogenous TGFβ activation by lung epithelial cells, which is the first report showing that caffeine can inhibit endogenous TGFβ activation in any cell type. Additionally, caffeine can interrupt fibroblastic profibrotic responses to TGFβ and these data show that caffeine inhibits TGFβ-induced increases in profibrotic genes including *PAI1, ACTA2* and *TGFB1.* This finding supports existing data from the liver showing that caffeine can interrupt profibrotic responses, particularly collagen and TGFβ expression in mesenchymal cells.^4–6^ Taken together, the data described highlight a potentially important role for caffeine and its analogues in the treatment of fibrotic lung disease.
