# Supplementary material for: Caffeine inhibits TGFβ activation in epithelial cells, interrupts fibroblast responses to TGFβ, and reduces established fibrosis in ex vivo precision-cut lung slices
Source: Thorax. 2016 Feb 24;71(6):565–7. doi: 10.1136/thoraxjnl-2015-208215 (PMC4893128; doi:10.1136/thoraxjnl-2015-208215)
Supplement: Supplementary data [file thoraxjnl-2015-208215supp.pdf]

<u>**Supplemental Material:**</u>

Methods

Cell Culture

In vitro cell experiments were performed on either lung epithelial cells or fibroblasts in submerged, monolayer culture. Immortalised human bronchial epithelial cells (iHBECs; gift from Professor Jerry Shay, University of Texas, USA) and primary human lung fibroblasts isolated from either non-fibrotic or IPF donors were used. iHBECs were cultured in keratinocyte serum free medium (KSFM; Gibco, UK) with 25µg/ml bovine pituitary extract (Gibco, UK), 0.2ng/ml recombinant epithelial growth factor (Gibco, UK), 250ng/ml puromycin (Sigma-Aldrich, UK) and 25µg/ml G418 (Sigma-Aldrich, UK). Fibroblasts were cultured in Dulbecco's modified Eagle's medium (DMEM; Sigma-Aldrich, UK) plus 10% foetal calf serum (PAA Laboratories, UK) and 4mM L-glutamine (Sigma-Aldrich, UK). All cells were growth arrested in unsupplemented media for 24 hours prior to experiments.

P-Smad2 ELISA

Levels of P-Smad2 in cell culture lysates were assessed using a commercially available P-Smad2 ELISA (Cell Signalling Technologies, UK). Briefly, cells were lysed in ice-cold lysis buffer (20mM Tris-HCl, 137mM NaCl, 1% Triton X-100, 2mM EDTA, 10% glycerol – all supplied by Sigma-Aldrich, UK) supplemented with protease and phosphatase inhibitors (Complete Mini protease inhibitor tablets and PhosStop tablets, Roche, UK). Protein concentrations were determined by bicinchroninic acid assay (Pierce, UK). 10µg total cell protein was used in the ELISA, which was performed according to the manufacturer's directions.

TGFβ Reporter Cell Assay

Transformed mink lung epithelial cells (TMLC) that stably express a TGFβ responsive region of the PAI1 gene driving luciferase were used as a reporter cell for TGFβ activity as previously described [9]. Fully confluent iHBECs or fibroblasts were co-cultured with 0.5 x 10^6 cells/ml TMLC and stimulated with increasing concentrations of caffeine for 16 hours. Cells were then lysed in luciferase assay system lysis buffer (Promega, UK) and luciferase activity was determined using a luminescence plate reader.

Quantitative Polymerase Chain Reaction (QPCR)

Expression levels of mRNA of *PAI1, ACTA2, TGFB1* and the housekeeping gene *B2M* (β2-microglobulin) were determined by QPCR as previously described for *PAI1* [9]. Briefly, following experimental testing cells were lysed in RA1 buffer (Macharey Nagel, UK) supplemented with 10µl/ml β-mercaptoethanol and total cell RNA isolated using Nucleospin RNA II isolation kit (Macharey Nagel, UK). RNA was reverse transcribed into complimentary DNA using murine Moloney leukaemia reverse transcriptase (Promega, UK) then subjected to QPCR analysis. Amplification was performed using an MXPro3000 (Stratagene, USA) with Kapa SYBR fast taq polymerase (Kapa Biosystems, UK) at an annealing temperature of 60°C. The primer sequences used were as follows: *PAI1* sense 5'-TCTGCAGACCTGGTTCCCAC-3', antisense 5'- AGCCCCGTAGTTCCATCCTG-3'; *ACTA2* sense GCTACGTGGGTGACGAAGCAC-3', antisense 5'-CATAATTTGAGTCATTTTCTCC-3'; *TGFB1* sense 5'-AAGGACCTCGGCTGGAAGTG-3', antisense 5'-CCCGGGTTATGCTGGTTGTA-3', *B2M* sense 5'-

AATCCAAATGCGGCATCT-3’, antisense 5’- GAGTATGCCTGCCGTGTG-3’. Amplification of a single DNA product was confirmed by melting curve analysis. Data was expressed as relative expression using the ΔΔCt equation as before [9].

MTT Assay

Cell and tissue viability was determined using a colorimetric MTT assay. For *in vitro* cell experiments, the cells were stimulated as required for the experiments then 200µg/ml 3-(4,5-dimethylthiazol-2-yl)-2,5-diphenyltetrazolium bromide (MTT; Sigma-Aldrich, UK) was added for one hour. Cell supernatants were aspirated and disposed of and the deposited formazan salts were dissolved in 200µl dimethyl sulfoxide (Sigma-Aldrich, UK). Absorbance was then measured at 595nm.

Induction of Pulmonary Fibrosis by Bleomycin

Animal studies were approved by the University of Nottingham ethical review committee and were performed under Home Office Project and Personal License authority within the Animals (Scientific Procedures) Act 1986. Animals received free access to food and water at all times. Male C57/black/6/J mice (Charles River, UK), aged 6-7 weeks, were treated with either 60IU bleomycin sulphate (Kyowa, Japan) in 50µl saline (Sigma-Aldrich, UK) or saline only as a control via the oropharyngeal route under isoflurane-induced anaesthesia (2.5%, 2L/min flow of oxygen). Animals were monitored over a 28 day period. After 28 days the animals were sacrificed by intraperitoneal overdose of pentobarbitone.

***Ex vivo* Precision Cut Lung Slice (PCLS) Model**

Following pentobarbitone overdose of mice the trachea was cannulated and the lungs were inflated with 1.3ml 2% low-melting point agarose (Sigma-Alrich, UK). 0.2ml of air was injected to displace the agarose from the airways in to the alveolar spaces and the lungs chilled to solidify the agarose. Once solidified, the agarose inflated lungs were removed and the lobes separated. PCLS (150µm thick) were prepared from all lobes using a Leica VT1200S vibrating microtome (Leica, UK) in Hank’s buffered saline solution (Sigma-Aldrich, UK) at 4°C. PCLS were equilibrated overnight at 37°C, 5% CO_2 in DMEM plus 200U/ml penicillin G and 200µg/ml streptomycin (both from Sigma-Aldrich, UK). The following day the PCLS were treated with increasing concentrations of caffeine (Sigma-Aldrich, UK) in DMEM, or DMEM only control, for 5 days. The PCLS were then blotted dry on tissue paper, weighed to determine tissue mass in mg, snap frozen in liquid nitrogen and stored at -80°C.

Assessment of Hydroxyproline by High Performance Liquid Chromatography (HPLC)

HPLC was utilised to measure hydroxyproline content of PCLS as previously described for fibroblasts [10]. Hydroxyproline was isolated and quantified by reverse-phase high-pressure liquid chromatography of 7-chloro-4-nitrobenzo-oxa-1,3-diazole. Hydroxyproline content was calculated by comparing peak areas from PCLS samples from the chromatogram with those from standard solutions (of known concentration) that were derivatised and separated under similar conditions on the same day.

Statistical Analysis

All *in vitro* cell experiments contained multiple technical repeats (minimum of two) and were performed a minimum of three independent times. Data from all three independent experimental repeats was amalgamated and shown in the figures. Errors bars denoted standard error of the mean (SEM). Statistical significance was determined by T test when comparing two data sets, Friedman's test for analysis of caffeine concentration-responses, or Kruskall-Wallis tests with Dunn's multiple comparison post-tests when comparing multiple data sets to analyse differences between specific groups. $P < 0.05$ was accepted as significant.
