# Supplementary material for: Caffeine inhibits TGFβ activation in epithelial cells, interrupts fibroblast responses to TGFβ, and reduces established fibrosis in ex vivo precision-cut lung slices
Source: Thorax. 2016 Feb 24;71(6):565–7. doi: 10.1136/thoraxjnl-2015-208215 (PMC4893128; doi:10.1136/thoraxjnl-2015-208215)
Supplement: Supplementary figure [file thoraxjnl-2015-208215supp_figure.pdf]

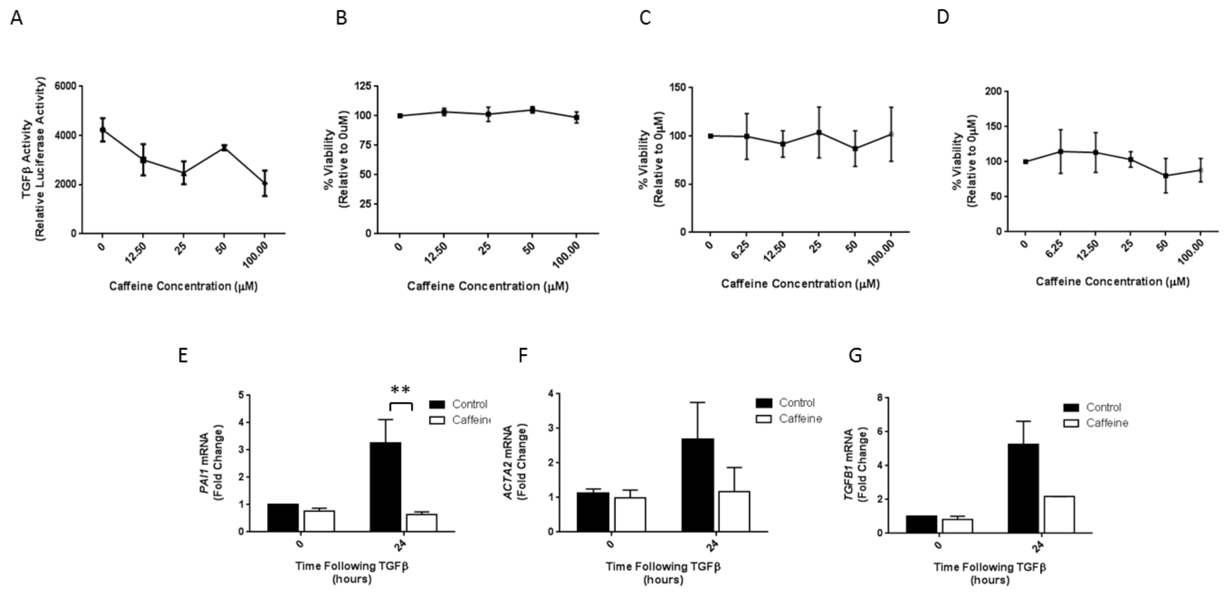


**Supplemental Figure**

A: iHBECs were stimulated with 0, 12.5, 25, 50 and 100µM caffeine and TGFβ activity measured by TMLC reporter cell assay. Caffeine reduced TGFβ activity in a concentration-dependent manner. Data are expressed as mean relative luciferase activity ± SEM from n=3 independent experiments.
B: iHBECs were stimulated with 0, 12.5, 25, 50 and 100µM caffeine and cell viability was measured by MTT assay. Caffeine had no effect on cell viability. Data are expressed as mean % viability ± SEM from 3 independent experiments.
C: NL fibroblasts were stimulated with 0, 6.25, 12.5, 25, 50 and 100µM caffeine and cell viability was measured by MTT assay. Caffeine had no effect on cell viability. Data are expressed as mean % viability ± SEM from experiments performed on cells from 3 individual donors.
D: IPF fibroblasts were stimulated with 0, 6.25, 12.5, 25, 50 and 100µM caffeine and cell viability was measured by MTT assay. Caffeine had no effect on cell viability. Data are expressed as mean % viability ± SEM from experiments performed on cells from 3 individual donors.

NL fibroblasts were pre-treated with 0 or 50µM caffeine for 30 minutes then stimulated with 0 or 2ng/ml TGFβ for 24 hours and E) *PAI1,* F) *ACTA2,* and G) *TGFB1* gene expression measured by QPCR. Data is expressed as mean fold change over control (0 hours, 0ng/ml TGFβ) ± SEM from experiments performed on cells from 3 individual donors

* p<0.05 **p<0.01
